# MoveONParkinson: developing a personalized motivational solution for Parkinson’s disease management

**DOI:** 10.3389/fpubh.2024.1420171

**Published:** 2024-08-19

**Authors:** Beatriz Alves, Pedro R. Mota, Daniela Sineiro, Ricardo Carmo, Pedro Santos, Patrícia Macedo, João Casaca Carreira, Rui Neves Madeira, Sofia Balula Dias, Carla Mendes Pereira

**Affiliations:** ^1^Escola Superior de Saúde, Instituto Politécnico de Setúbal, Setúbal, Portugal; ^2^Escola Superior de Tecnologia, Instituto Politécnico de Setúbal, Setúbal, Portugal; ^3^Research Center for Engineering and Sustainable Development (SUSTAIN), Setúbal, Portugal; ^4^NOVA Laboratory for Computer Science and Informatics (NOVA LINCS), NOVA University Lisbon, Lisbon, Portugal; ^5^NOVA School of Science and Technology, Center of Technology and Systems (UNINOVA-CTS) and Associated Lab of Intelligent Systems (LASI), NOVA University Lisbon, Lisbon, Portugal; ^6^Comprehensive Health Research Centre (CHRC), NOVA University Lisbon, Lisbon, Portugal; ^7^Interdisciplinary Centre for the Study of Human Performance (CIPER), Faculdade de Motricidade Humana, Universidade de Lisboa, Lisbon, Portugal

**Keywords:** Parkinson’s disease, mobile health, MoveONParkinson, self-efficacy, user engagement, self-management, behavior change

## Abstract

**Introduction:**

Despite the effectiveness of exercise-based interventions on symptom management and disease progression, many people with Parkinson’s Disease (PwPD) do not exercise regularly. In line with the ubiquitous use of digital health technology, the MoveONParkinson digital solution was developed, comprising a Web Platform and a Mobile App with a Conversational Agent (CA). The interface features were designed based on the principles of Social Cognitive Theory with the goal of fostering behavior change in PwPD for sustained exercise participation and improved disease management.

**Methods:**

Using a mixed methods approach, this study aimed to collect feedback, assess the acceptability of the Mobile App and the Web Platform, and evaluate the usability of the latter. Quantitative data, which included questionnaire responses and the System Usability Scale (SUS) scores, were analyzed using descriptive statistics, heatmaps, and correlation matrices. Qualitative data, comprising semi-structured and thinking-aloud interview transcripts, were subjected to an inductive thematic analysis. A total of 28 participants were involved in the study, comprising 20 physiotherapists (average age: 34.50 ± 10.4), and eight PwPD (average age: 65.75 ± 8.63; mean Hoehn & Yahr: 2.0 (± 0.76)).

**Results:**

Three main themes emerged from the thematic analysis of the interviews, namely: Self-management (Theme 1), User Engagement (Theme 2), and Recommendations (Theme 3). The assessment of the Mobile App and the CA (mean score: 4.42/5.0 ± 0.79) suggests that PwPD were able to navigate this interface without notable difficulties. The mean SUS score of 79.50 (± 12.40%) with a 95% confidence interval ranging from 73.70 to 85.30, reveal good usability.

**Discussion:**

These findings indicate a high level of acceptability of the MoveONParkinson digital solution, serving as a foundation for assessing its impact on exercise engagement and, subsequently, its influence on symptom management and quality of life of PwPD.

## Introduction

Neurological disorders are the current leading source of disability, and Parkinson’s Disease (PD) is the most common and fastest movement disorder growing in prevalence and disease burden (1). Fueled by population aging and increasing longevity, the number of people with PD (PwPD) is projected to exceed 12 million worldwide by 2040, referred as the Parkinson pandemic (2).

As a neurodegenerative disease, physical activity and exercise-based physiotherapy interventions have been highlighted for its effectiveness on PD management, particularly by improving motor and non-motor symptoms and quality of life (QoL) (3, 4). Nonetheless, many PwPD do not comply with exercise guidelines. Besides environmental barriers (i.e., lack of accessibility, financial burden) and PD non-motor symptoms, the lack of exercise engagement is greatly attributed to personal beliefs of PwPD, namely low outcome expectations and low self-efficacy (5, 6). Hence, preventing a cycle of inactivity and promoting long-term engagement in socially supported activity is a priority for PwPD in the self-management of their condition (7). Moreover, it is recommended to incorporate interventions that include self-management support into rehabilitation, as they contribute to informed and motivated individuals, leading to better outcomes, including the slowing of disease progression, reduction of complications, and cost savings, ultimately supporting PwPD in maintaining their independence and enhancing their overall QoL (7–9). The development of interventions for PD management should adopt a personalized care management approach, aiming to empower individuals with PD, while also implementing recognized strategies to achieve the Quadruple Aim, a model for integrated and patient-centered care in PD (10, 11). Hence, supported behavior change interventions addressing self-management need to be PD specific for the target behavior to be initiated and maintained at a clinically meaningful level, since the progressive nature of the disease and its non-motor symptoms present both physical and psychosocial challenges (7, 12). In this context, the use of established behavioral theories in the design, implementation, and evaluation of interventions has been suggested to enhance behavior change (13).

Moreover, digital interventions based on the Social Cognitive Theory (SCT) for self-management of several chronic conditions and health domains, including exercise and physical activity, has been highlighted in the literature (14–17). For instance, a digital intervention for PD management grounded by the constructs of the SCT has been developed, comprising a mobile interface with an integrated ParkinsonBot Conversational Agent (CA), aiming to respond to questions about PD posed by PwPD and their caregivers (18–20). Indeed, this approach has been reinforced by recent evidence demonstrating the effectiveness of CAs rooted in behavioral theory for facilitating self-management of chronic diseases and implementing lifestyle change interventions, with the added benefit of being well-received by users and proven to enhance patient outcomes (21). In this way, the ubiquitous use of digital health technology presents major opportunities for improving QoL of individuals with neurodegenerative diseases, namely trough the development of digital interventions for health-related behavior change (22–24). In fact, several studies have also assessed digital interventions involving mobile health (mHealth) apps aiming to support exercise engagement in PwPD (25–29). Despite these interventions containing key elements for facilitating behavior change (i.e., remote monitoring, personalized exercise, behavior change techniques, gamification), intervention design was not grounded by behavioral theory. Conversely, the evaluation of a mHealth intervention for PD self-management grounded by the Information-Motivation-Behavioral skills model delivered promising results on self-efficacy and non-motor symptoms (24). In addition, a recent systematic review aiming to explore the features and characteristics of mobile apps for self-care in PwPD underlined the need to engage in quality research for the development and evaluation of mHealth solutions as useful and reliable tools for PwPD and healthcare professionals (HCPs) (23).

Inspired by the considerations above, this paper introduces MoveONParkinson, an innovative digital solution designed for personalized, home-based exercise, with its foundations rooted in the constructs of the SCT. This digital solution is designed to empower PwPD, motivating them to embrace a specific target behavior, specifically, engagement in exercise, which they can execute effectively, with the ultimate goal of improving PD management. With this aim in mind, the present research study evaluates the interfaces of the MoveONParkinson digital solution, comprising web and mobile interfaces, with a specific focus on gathering feedback and assess the acceptability of the Mobile App, including its Conversational Agent, and to gather feedback and assess the acceptability and usability of the Web Platform.

## Theoretical background

### Health behavior change theories

As a digital health behavior change intervention, MoveONParkinson solution was grounded by the Integrate, Design, Assess, and Share (IDEAS) framework. Envisioning several aspects of design thinking and user-centered design, its main strengths comprise the involvement of stakeholders throughout the development process and the iterative approach ensuring its responsiveness to changing contexts (30). The IDEAS framework suggests drawing intervention design on behavioral theory, which enhances the effectiveness of behavior change (13). In addition, supported behavior change must be provided to sustain new health behaviors, as without it the transition from health care provision to true self-management is unlikely (12).

The most frequently used theories in health behavior research are the SCT, the Health Belief Model, and the Transtheoretical Model (30). Nonetheless, the SCT is one of the most widely used models to explain and improve self-management in patients with chronic illnesses. It has been applied to the initiation of personal behavior change and the maintenance of the acquired behavior (31), including in mHealth interventions (30). According to this theory, individual behavior is determined by the interaction between cognitive, behavioral, and socioenvironmental influences (32, 33). Self-efficacy, the individual’s belief in their capability to perform a specific behavior and achieve desired outcomes, along with social support, which entails the perception of encouragement from one’s social network, are fundamental components of the SCT that play a pivotal role in influencing self-management and behavior change (34). Higher levels of self-efficacy increase the likelihood of a successful performance, with outcome expectations, socio-structural factors, and goal setting as intermediate variables toward the maintenance of health behaviors. Considering exercise engagement as the target behavior, self-efficacy reflects the individual belief of successfully engaging in exercise and is a stronger predictor of exercise engagement than disease severity (32–36).

### Acceptability assessment frameworks

Within the IDEAS framework, this study primarily focuses on assessing the acceptability of MoveONParkinson. More specifically, acceptability concerns to individuals’ affective attitudes, usage intentions, actual usage, and post-engagement satisfaction with new digital health interventions (37). In fact, enhancing acceptability involves iterative cycles based on user feedback to enhance intervention usability, and ensure it enables safe, effective, and efficient task performance, contributing to a positive user experience (38).

Several frameworks have been developed aiming to assess the acceptability of digital health interventions. In particular, the Theoretical Framework of Acceptability is a comprehensive framework that reflects the extent to which people delivering or receiving a technology-based intervention consider it to be appropriate, based on cognitive and emotional responses. It comprises seven domains, namely: (1) affective attitude; (2) burden; (3) ethicality; (4) intervention coherence; (5) opportunity costs; (6) perceived effectiveness; and (7) self-efficacy (39, 40). In addition, the Unified Theory of Acceptance and Use of Technology (UTAUT) is based on an analysis and comparison of technology acceptance models, aiming to assess the likelihood of acceptance of new technologies and to capture the determinants for user acceptance. It comprises four key variables: (1) performance expectancy (degree of belief that using the technology will help increase performance); (2) effort expectancy (degree of ease associated with using the technology); (3) social influence (degree to which a user is influenced by the opinions of significant others who believe that they should use the technology); and (4) facilitating conditions (degree to which using the technology will be supported at an organizational level) (41). Furthermore, the Technology Acceptance Model (TAM) is based on the principles of the Theory of Reasoned Action, hypothesizing that users’ perceived ease of use (the degree to which the user expects the system to be free of effort) and perceived usefulness (perspective of the user that using the system will increase their performance) provide insights into their behavioral intentions (willingness of the user in exerting effort to perform the target-behavior) toward acceptance of the technology (42).

Motivated by both the widespread use of SCT (30) and TAM constructs (42), we explored various aspects that assess the acceptability and usability of the proposed MoveONParkinson solution, as reflected in identified themes from the qualitative analysis and the related metrics from the quantitative analysis, respectively, described in detail in the Results section. This study contributes significantly to the knowledge on PD management and digital health interventions. By integrating SCT into the MoveONParkinson solution, it provides a robust foundation for promoting exercise engagement among PwPD. The MoveONParkinson solution offers personalized home-based exercise tailored to individual needs, which is crucial given the progressive nature of PD. In addition, the present study employs comprehensive acceptability assessment frameworks, including the Theoretical Framework of Acceptability, UTAUT, and TAM, to evaluate user perceptions and attitudes. A novel aspect of the study is the inclusion of the ParkinsonBot CA, which helps facilitate self-management and behavior change. The focus on self-efficacy and social support highlights their importance in influencing exercise engagement and self-management, aligning with SCT principles. Additionally, the study addresses the needs of HCPs and caregivers, highlighting how digital tools can optimize treatment plans and provide better support, promoting a more integrated and patient-centered care approach.

## Materials and methods

### The MoveONParkinson digital solution

**Figure 1:**
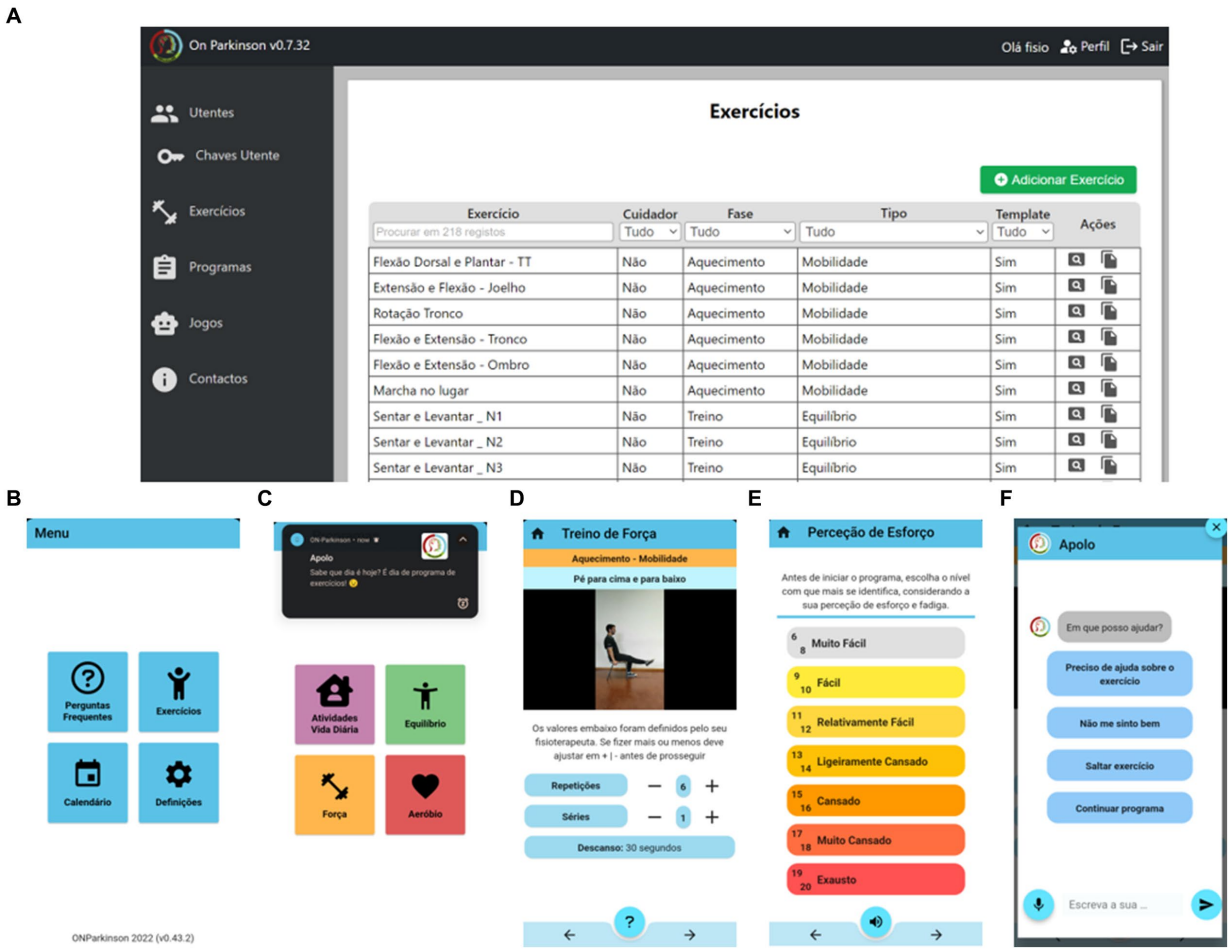
Screenshots of the MoveONParkinson digital solution. **(A)** Exercise database of the Web Platform, which menu comprises “Patients,” “Patients’ Passwords,” “Exercises,” “Programs” and “Contacts”; **(B)** Front page of the MoveONParkinson mobile app; **(C)** Exercise Module of the mobile app, with a motivational cue provided the Conversational Agent integrated within the mobile app; **(D)** Video, audio instructions, and exercise prescription within an exercise program; **(E)** Measurement of Rate of Perceived Exertion (43), which is required both at the beginning and the end of each exercise program; **(F)** Main screen of the Conversational Agent.

The interfaces of the MoveONParkinson digital solution encompass a Web Platform, designed to optimize therapy outside clinical settings, offer remote support by HCPs, and prescribe exercise for PwPD, as well as a mobile app that targets the self-management of PwPD and caregivers (CGs), providing access to reliable PD information, facilitating communication with HCPs, and offering personalized exercise programs (see Figure 1). Informed by SCT (32, 33), MoveONParkinson incorporates: (i) the behavioral aspect, focusing on mastering experiences and achieving successful performance through personalized exercise and information delivery via the Conversational Agent (CA); (ii) addressing barriers using motivational strategies like persuasion, encouragement, and emotional reinforcement, which include features like exercise reminders and messages of positive reinforcement from the CA, offering exercise programs that can be performed at home with everyday items, and providing free app access; (iii) reinforcing facilitators such as exercising with peers, including CGs in exercise programs, and facilitating vicarious learning through exercise videos; and (iv) cognitive aspects incorporated in the Frequently Asked Questions module of the App. Within the MoveONParkinson Web Platform, goals are set in accordance with the physiotherapist, which are then transposed to the digital interfaces for follow up. Action planning also takes place in a clinical setting, to mitigate possible barriers for exercise. Problem solving is discussed with the physiotherapist, but also reinforced through the CA that guides users through the exercise programs by answering their questions. Self-monitoring is encouraged through the MoveONParkinson Mobile App, where PwPD are able to follow up on their progress regarding daily physical activity and exercise performance.

The MoveONParkinson digital solution was developed based on recent guidelines for PD management, which highlight the relevance of implementing exercise programs for aerobic conditioning, strength, balance, and Activities of Daily Living (ADLs) (3, 44–49). In order to cover the variability in the PD severity, a stream of exercises with a layer of PD specificity was included, particularly for balance training and ADLs, where many PwPD experience significant impairments. Moreover, in an inclusive perspective, more aerobic-based general exercises were also included, as most strength and aerobic exercises follow straightforward movement patterns to facilitate being performed at home, either with caregiver supervision or alone. The MoveONParkinson Web Platform (see Figure 1A) provides an overview of the database of exercises. The exercises uploaded to this interface comprise written instructions (i.e., commands, safety precautions, equipment) and exercise videos with audio description. This allows physiotherapists to develop and assign exercise programs to PwPD, who then access the latter using the MoveONParkinson mobile app (see Figures 1B–F). The exercise programs of the MoveONParkinson Web Platform can be personalized in accordance with the needs of each PwPD. There are some general exercise templates available on the platform, to provide guidance regarding the type of personalization that can be achieved. However, physiotherapists can create their own exercise programs for each patient and update them accordingly. Afterwards, performance feedback is provided, which allows physiotherapists to modify the program and PwPD to access their exercise history. In this vein, the physiotherapist can have access to the metrics of the exercise program, particularly regarding the prescription parameters (e.g., how many sets/repetitions were performed in relation to the ones that were prescribed, what was the duration of the exercise, what was the perceived intensity from the patient), and can be able to access biometric data (i.e., heart rate). In its current version, the MoveONParkinson mobile app does not provide specific metrics as feedback to the user (PwPD). Nevertheless, in the next version (currently under development), the mobile app will provide feedback in a simpler representation of the metrics provided to the physiotherapists through the Web Platform, i.e., overview vs. detailed information. Moreover, future research will focus on incorporating users’ responses to generate more dynamic replies from the CA, which should be based on a Large Language Model like ChatGPT.

A CA integrated in the MoveONParkinson mobile app (see Figure 1F) was also designed to address barriers for exercise engagement by providing assistance to PwPD during exercise programs. The CA is built upon standardized questions mainly related with the exercise program, namely, to clarify concepts related with exercise prescription, to report issues during the program, as well as being able to pause or stop the program before its completion. Moreover, the CA is able to answer questions from the user, as long as the questions are in its domain of knowledge (e.g., PD management-related aspects, exercise programs). Then, the user can either select one of the responses from the menu or write an open-ended response. The role of the CA is extended in the field of reinforcements/reminders; in fact, the latter are always provided when the user opens the app, when they start an exercise program and upon its completion. Further reinforcements will be delivered as daily notifications, that can be adjusted depending on the level of adherence from the user.

### Study design

A mixed methods convergent approach (50, 51) was used to obtain complementarity on the process of gathering feedback and assessing the MoveONParkinson digital interfaces. Quantitative and qualitative data were integrated using an interactive approach for the study design, and a weaving narrative for data reporting, which was further complemented with a joint display (52–54).

### Sample and recruitment

Recruitment occurred between June and October 2022. A purposive sample was used to maximize the possibility of obtaining accurate and detailed feedback. PwPD were invited to participate through a physiotherapist affiliated with the Portuguese Association of Parkinson’s Disease (APDPk), who took into account the following criteria: PwPD aged ≥18 years were included if presenting: (a) clinical diagnosis of PD; (b) the ability to understand the study aim and to compromise with the required tasks; (d) the capacity to provide written informed consent. PwPD were excluded if presenting: (a) severe vision or hearing impairments; (b) a diagnosis of severe neuropsychiatric symptoms (i.e., major depression); (c) severe cognitive or physical impairment (Hoehn and Yahr stage 5). Physiotherapists working with PwPD were also invited to participate in the study. Participants did not receive any reward during the recruitment process or for their contribution to the data collection.

Using G*Power 3.1.9.7 tool, the power analysis was conducted by applying Wilcoxon-signed rank test with an effect size of *d* = 0.5 [that approximates the average size of observed effects in various fields (55)], probability error of *p* = 0.05, and power of 0.80, resulting in a total sample size of 28, (noncentrality parameter *δ* = 2.85, critical *t* = 1.70, and degrees of freedom Df = 25.73). The estimation of power in this acceptability study is suggested to explore the effect of type II error rate, as studies with low statistical power (high type II error) can also lead to increase the type I error rates (56). This study aimed to gather feedback and assess the acceptability of the MoveONParkinson Mobile App and the CA, and assess the acceptability and usability of the Web Platform, considering all participants as one user group under the co-creation approach. Presenting the results (qualitatively and quantitatively) for the PwPD and physiotherapists separately provides us with some focus on the way the feedback was conveyed by different perspectives, the one of the PwPD side and the other from the physiotherapists. Since we have already pre-described the two subgroups in our study design, there is no data-driven biases, increasing the reliability of the findings (57).

### Data collection

Detailed information about the study aims and procedures were provided to the participants who were eligible and demonstrated interest in enrolment, after which they provided informed consent.

A socio-demographic background questionnaire, including physical activity and exercise levels, details on the use of technology in daily living, and clinical data was used for PwPD. PD staging was reported according to the Hoehn and Yahr Scale (H&Y). The assessment of the MoveONParkinson Mobile App and the CA took place at APDPk, lasting from 30 to 40 min. PwPD were introduced to the key features of the interface through a demonstration provided by the researcher (first author, BA), participants had a 5-min period to explore the app without specific instructions. Then, they were asked to select the icons Exercises>Programs>Strength (see Figure 1). Participants were encouraged to read the instructions, play the videos, listen to the corresponding audio, and to classify their Rate of Perceived Exertion at the beginning and the end of the exercise program (despite not physically following it). Then, PwPD experienced the features of the CA (i.e., writing, reading, speaking, listening, and selecting questions), which were evaluated using an online questionnaire, consisting of 12 questions with the answer options as a 5-point Likert scale based on strength of agreement (see Supplementary material—Online questionnaire). Then, PwPD provided additional feedback through semi-structured interviews, which were audio recorded (see Supplementary material—Patient Interview Guide Script).

In addition, a socio-demographic background questionnaire, including questions regarding clinical practice and frequency of exercise prescription was used for physiotherapists. The usability tests of the MoveONParkinson Web Platform, where end-users work through scenarios representing usage of the interface, were conducted with physiotherapists, separately than the sessions with PwPD, using Zoom platform, and were audio and video recorded. The session lasted between 45 and 60 min. Participants were introduced to the key features of the interface through a video and audio tutorial, after which they had a 5-min period to freely explore the platform. More specifically, the tasks included: (a) add an exercise; (b) view and edit an exercise; (c) create an exercise program; (d) assign the program to a patient; and (e) review the exercise-related data of PwPD. Physiotherapists were asked to verbalize their thoughts throughout this process using the thinking aloud method (58, 59), and provided additional feedback through semi-structured interviews (see Supplementary material—Physiotherapist Interview Guide Script).

Moreover, the usability of the MoveONParkinson Web Platform was evaluated using the System Usability Scale (SUS), a validated tool for European Portuguese (60). The SUS comprises 10 statements scored on a 5-point Likert scale of strength of agreement, and its final score ranges from 0 to 100. A score exceeding 80 indicates a high level of usability, signifying that users are likely to recommend the product to others, while 68 points represent the 50th percentile, and 70 points indicate the acceptability threshold (61–63).

### Data analysis

Thinking aloud and semi-structured interviews were transcribed verbatim using Microsoft Office Word. All qualitative data was analyzed by two independent researchers (BA, CMP) following an inductive approach for thematic analysis. Initial codes were developed through a comprehensive data analysis process. The transcripts were then reviewed to assess code reliability. Once generating the themes, these were reviewed based on the criteria of internal homogeneity and external heterogeneity and a thematic map was attained (64, 65).

Quantitative data were exported from Google Forms to Microsoft Office Excel. The SUS scores were calculated and the confidence interval (CI) for the mean score was calculated with a 95% confidence level (66). The SUS scores, the answers regarding the CA and socio-demographic data were analyzed using the descriptive statistic measures of mean, standard deviation, and range. The relationships between SUS scores and socio-demographic characteristics of physiotherapists, as well as between the assessment of the CA and socio-demographic characteristics of PwPD, were quantified and summarized using correlation matrices. A higher correlation coefficient indicates a stronger relationship. Positive correlations suggest a tendency for higher scores with specific socio-demographic attributes, while negative correlations indicate an inverse relationship. A circular heatmap was used to investigate the response patterns regarding the CA assessment and the SUS, where each cell is color-coded to represent the value of the response. A dendrogram complemented the latter, aiming to perform a hierarchical clustering analysis of the data, where the height at which branches merge provides information about the dissimilarity between clusters, guiding the identification of subgroups based on answer similarity.

Finally, in order to facilitate the integration of the data, a joint display was used to draw relationships between the qualitative and quantitative constructs. This approach aimed to systematically restructure the findings, thereby optimizing the presentation of the data (52–54).

## Results

A total of 35 individuals were initially contacted to enroll in the research study, consisting of 25 physiotherapists and 10 PwPD. Ultimately, 28 individuals accepted the study invitation and underwent eligibility screening. The final sample included 20 physiotherapists (mean age: 34.50 ± 10.47 years; 12 males) and eight PwPD (mean age: 65.75 ± 8.63 years; mean H&Y of 2.0 ± 0.76; H&Y range: 1–3; median H&Y: 3; 5 males). Further sociodemographic information of the study sample is detailed in Table 1. In this study, we have included PwPD ranging from H&Y stages 1–3, covering mild to moderate disease severity. Despite H&Y stage 5 being an exclusion criterion due to the severity of impairments, no participants even in H&Y stage 4 were considered for inclusion, due to the purposive sampling method. Regarding the experience of PwPD with mHealth apps, six (75%) participants have never used similar apps to this Mobile interface, and one participant had previously used an app for exercising. Within the 19 physiotherapists currently working with patients, 11 (57.89%) reported prescribing exercise frequently, five (26.32%) occasionally, and three (15.79%) rarely. As to exercise prescription for PwPD, 10 physiotherapists (52.63%) prescribe frequently, two (10.53%) occasionally, six (3158%) rarely, and one (5.26%) never prescribes exercise for PwPD.

**Table 1:** Sociodemographic and clinical information of PwPD and physiotherapists.

PwPD (*n* = 8)	Age (years)	65.75 ± 8.63
	Gender (male)	5 (62.5%)
	Time since diagnosis (years)	11.63 ± 6.80 (0–21)
	Hoehn and Yahr score	2.0 ± 0.76 (1–3) Stage 1: 2 (25%) Stage 2: 4 (50%) Stage 3: 2 (25%)
	Educational background	5–9 years of school: 3 (37.5%) 10–12 years of school: 4 (50%) University education: 1 (12.5%)
	Professional status	Retired: 7 (87.5%) Employed: 1 (12.5%)
	Physiotherapy attendance	Yes: 7 (87.5%) No: 1 (12.5%)
	Physiotherapy weekly frequency	Twice: 3 (43%) Three times: 4 (57%)
	Exercise engagement outside the clinical setting	Yes: 5 (62.5%) No: 3 (37.5%)
	Use of digital devices	Smartphone: 6 (75%) Tablet: 1 (12.5%) Computer: 5 (62.5%)
Physiotherapists (*n* = 20)	Age (years)	34.50 ± 10.47
	Gender (male)	12 (60%)
	Educational background	Bachelor’s (Hons) Degree: 11 (55%) Post-graduation: 6 (30%) Masters: 2 (10%) PhD: 1 (5%)
	Clinical practise (years)	10.74 ± 9.17 (1.5–38)
	Experience in neurology (years)	9.84 ± 9.30 (0.5–38)

### Qualitative analysis findings

From the thematic analysis three main themes emerged regarding the Mobile and Web interfaces of the MoveONParkinson digital solution, namely: Self-management (Theme 1); User Engagement (Theme 2); and Recommendations (Theme 3). In the following subsections, please note that participant groups (i.e., PwPD, Physiotherapist) are denoted within quotation marks, along with an indication of the interface under discussion (i.e., Web Platform, Mobile App).

### Self-management (Theme 1)

Overall, PwPD reinforced the role of the MoveONParkinson Mobile App on patient empowerment, by providing resources to work through their issues, independently. From their perspectives, this interface may be considered a suitable tool for exercise engagement, by demonstrating the exercises and providing clear instructions. The role of the mobile app in giving patients control over their lives (i.e., exercising at their own convenience, actively engaging in rehabilitation) was also emphasized by several physiotherapists. According to their perspectives, the subsequent increase in autonomy leads to reduced dependence on HCPs, who should foster a collaborative approach to disease management. It was also recognized that patient autonomy surpasses exercise performance, also impacting psychological and cognitive dimensions, which were considered vital for shifting patient care toward self-management. In addition, this interface was considered particularly relevant for PwPD in early disease stages and for those with an active lifestyle, to provide support throughout the disease course. These perspectives are outlined below:

"*I believe the MoveONParkinson App offers numerous advantages as it provides clear exercise instructions and it is easy to identify which exercises to perform. While some minor corrections may be needed, they are relatively insignificant.*" (PwPD#8, Mobile App)

"*The use of the MoveONParkinson App increases patient autonomy. We gain the ability to find solutions through the available resources and address our issues independently.*" (PwPD#2, Mobile App)

"*Solutions of this kind are crucial in empowering patients control over their own lives. They can exercise at their convenience, actively engaging in their rehabilitation. This autonomy offers numerous benefits, such as reducing their dependence on healthcare professionals. This is vital because sometimes we inadvertently rule patients' lives when they should feel empowered and in control. (…) If the patient can independently complete the exercise program, it fosters patient autonomy, which is highly significant to effectively promote self-management.*" (Physiotherapist#11, Mobile App)

"*I consider the mobile app a crucial tool for people with PD. We have patients who still maintain a certain degree of independence and an active lifestyle, and for those at early PD stages, this app can serve as valuable support.*" (Physiotherapist#1, Mobile App)

Regarding the MoveONParkinson Web Platform, some physiotherapists mentioned that it could be a valuable tool for bridging the gap between in-person care and telerehabilitation. In particular, acknowledging the common decline in motivation among PwPD and giving priority to their safety, a phased approach, moving from in-person care to telerehabilitation, was recommended. This approach involves initiating in-person assessment and intervention design and testing before exploring telerehabilitation. Additionally, some physiotherapists found the MoveONParkinson Web Platform valuable for customizing exercise-based interventions for PwPD, with a focus on personalized exercises and adaptable programs based on patient feedback. Also, the limited availability of innovative resources such as the MoveONParkinson Web Platform for targeting the needs of patients with neurological conditions, particularly PwPD was outlined by one participant, as follows:

"*The MoveONParkinson Web Platform may have potential for bridging the gap between one-on-one, personalized care and telerehabilitation. Initially, I would conduct a face-to-face patient assessment, design an intervention program, and the patient would then undergo in-person testing of the program. (…) We must not overlook patient motivation, which tends to decline over time, especially given the mobility and cognitive function impairments. Individualization and self-reliance are not typical traits of populations with basal ganglia lesions.*" (Physiotherapist#1, Web Platform)

"*While there are platforms designed for creating and delivering exercises to patients, the MoveONParkinson Web Platform allows to modify programs based on real-time feedback, and to verify whether patients have achieved their goals, which is much more relevant.*" (Physiotherapist#12, Web Platform)

"*In the field of neurology, there is limited availability of these resources. While there are some older programs primarily designed for conditions like stroke and spinal cord injury, they are often outdated. Moreover, since these only serve a basic purpose of exercise selection and delivery, they are entirely distinct from the capabilities of the MoveONParkinson Web Platform.*" (Physiotherapist#20, Web Platform)

### User engagement (Theme 2)

From the perspectives of PwPD, while the MoveONParkinson Mobile App was found suitable for this patient population, the necessary commitment to engage in their rehabilitation was outlined. Specific features of the App were highlighted as facilitators for user engagement with this interface, particularly the clear and concise exercise instructions, regular app usage, and prior familiarity with the exercises. Regarding the impact of social interaction on motivation, some PwPD outlined that exercising with company or supervision is particularly encouraging. In this line, several physiotherapists noted the benefits of involving CGs, not only for strengthening the relationship between PwPDs and CGs, but also to increase their physical activity levels. In addition, the relevance of a collaborative approach for PD management was outlined by some physiotherapists, where HCPs provide guidance to PwPD through a clear process of goal setting. Additional facilitators were reported, namely overcoming of need for a particular time and place to exercise, and the minimal equipment required to safely perform a structured home-based exercise program. On the contrary, participants identified several barriers for engagement with the MoveOnParkinson Mobile App. These included a preference for supervised exercise within a clinical setting, and a lower level of digital health literacy. In this line, the perspectives of some participants are outlined below:

"*I believe the MoveONParkinson App is suitable for people with Parkinson's, but it requires the patient's willingness to engage in the exercises. The exercise descriptions within the app are detailed enough for clear understanding. I have been familiar with the demonstrated exercises for a long time. While these may appear new to beginners, regular use of the app will enable them to follow along.*" (PwPD#1, Mobile App)

"*I enjoyed using the Mobile App, and exercising with company or under supervision is always more motivating.*" (PwPD#7, Mobile App)

"*The patient receives guidance from a healthcare professional who outlines the goals to be achieved. This partnership, with the patient's belief and the healthcare professional bridging the gap, can lead to successful engagement with the program.*" (Physiotherapist#19, Mobile App)

"*The most significant advantage is that there is no need for a specific time or place to exercise, it completely overcomes spatial and time barriers. (…) Patients do not require much equipment to perform a well-structured and safe program at home. Involving the caregiver not only fosters a stronger relationship between the dyad but also contributes to the caregiver's physical activity. The app could pose a challenge for those who may not be as comfortable with technology, particularly among the older generation. While our population is becoming increasingly educated on technology, there are still individuals who have limited access. (…) While some patients might feel at ease with this approach, there are others who prefer the presence of a healthcare professional during rehabilitation.*" (Physiotherapist#7, Mobile App)

Regarding the MoveOnParkinson Web Platform, many physiotherapists outlined an efficient and simple design, that quickly enables them to create an exercise or program, as the main facilitator for user engagement. Conversely, one physiotherapist identified the constraints imposed by rehabilitation setting policies (i.e., facilities do not actively encourage patients to decrease in-person physiotherapy sessions) as a barrier for user engagement with this interface. In addition, some physiotherapists highlighted the initial time commitment needed to become familiar with an extensive exercise database, and the relevance of having an organized database of exercises to increase searching efficiency. To illustrate these viewpoints, two physiotherapists specifically stated the following:

"*The primary advantage lies in the ease of creating exercise programs. Even creating individual exercises is straightforward. After three minutes exploring, I was already able to create a program or an exercise. I did not find it complicated to work with the Web Platform.*" (Physiotherapist#4, Web Platform)

"*The primary drawback doesn't necessarily lie with the physiotherapist, but rather with the organization of the services. These may not encourage patients to discontinue their in-person exercise sessions, which can pose a barrier. Convincing people to make this transition can be quite challenging. (…) To be able to use the Web Platform effectively, I must be aware of its contents. Imagine the time I would have to invest, and, at the same time, potentially lose, if I had to review each exercise. With a database of 200 exercises, it can become challenging to navigate without some level of categorization.*" (Physiotherapist#2, Web Platform)

### Recommendations (Theme 3)

Concerning the MoveOnParkinson Mobile App, most PwPD did not encounter major difficulties while using this interface. Nonetheless, it was recognized that more comprehensive feedback might emerge with regular app usage, since the current assessment only included reading through the exercise program, without performing the exercises. Once the assessment of the MoveOnParkinson App was completed, some PwPD suggested including images alongside exercise descriptions and noted the need for clearer audio descriptions in exercise videos (i.e., slower playback speed, similar to the pace of the CA voice). In addition, some physiotherapists recommended incorporating relatable metrics within the app to enhance motivation. For instance, one physiotherapist suggested providing measurable data that can visually demonstrate the outcomes of accomplishing exercise-related goals (i.e., walking distance per day), thus providing evidence of patient progress. Moreover, other physiotherapists noted that since not all PwPD possess similar cognitive capabilities, exercise descriptions should be as clear and comprehensive as possible. Particularly attending to PwPD with cognitive impairment, the narrator should be mindful of their voice tone and pace since a lack of comprehension could become a source of distress. In this context, perspectives of participants are outlined as follows:

"*Currently, I don't have the space or time to say that I don't face any difficulties with the app. Undoubtedly, some challenges may arise with regular use.*" (PwPD#2, Mobile App)

"*If patients could see metrics like 'you have covered X kilometers and received Y benefits related to a particular health parameter, ' I believe it would be highly motivating. This way, patients can visually track the benefits of their exercise efforts.*" (Physiotherapist#15, Mobile App)

"*Despite the presence of images on the App, the clarity of video explanations is highly relevant. It is essential to focus on a careful articulation of exercise instructions, using language that is clear, technically sound, and accessible, all the while maintaining a relaxed tone. This is particularly vital for patients with cognitive impairments. They already face numerous challenges, and any element they may not comprehend can become a source of distress.*" (Physiotherapist#16, Mobile App)

In line with aforementioned recommendations for improving the MoveONParkinson Web Platform, several physiotherapists suggested incorporating multimodal exercise programs (i.e., strength, aerobic, balance, ADLs), taking into consideration the preferences of PwPD and symptom management. In addition, some physiotherapists suggested increasing the efficiency of exercise selection by associating an image with the exercise name and developing search filters (i.e., training phases, equipment, type of exercise, individual exercise or in pairs). One physiotherapist suggested including template notes in the available text boxes (i.e., reminders to attend breathing patterns) to make the process of creating exercise programs less time-consuming. Some design-related recommendations were also provided to ensure a more intuitive navigation within this interface. In addition, several physiotherapists outlined the importance of accessing clinical and exercise data in each patients’ profile (i.e., medical complications, falls) to monitor patient progress and better tailor interventions according to their needs. As examples of these perspectives, three physiotherapists highlighted the following:

"*It makes more sense when exercise types can be combined within the same program, making the experience more enjoyable for patients. It does not seem logical not to use hybrid programs because we often need to address multiple components. For instance, when dealing with a patient experiencing significant fatigue, it is more practical to incorporate various exercise types to prevent excessive fatigue in specific muscle groups or energy systems. I see this as a potential improvement.*" (Physiotherapist#10, Web Platform)

"*Selecting exercises from the database is time-consuming. It would be helpful if there was an image associated with each exercise. (…) Including filters for caregiver presence, training phase, and required equipment would facilitate exercise selection. It would be more efficient having suggested template notes, such as reminders for patients to pay attention to their breathing, to stay hydrated, or to take breaks between exercises. These would spare me the need to write. There are also aspects in need of design improvements, this Web Platform could have a more user-friendly and intuitive design.*" (Physiotherapist#19, Web Platform)

"*When accessing the patient's profile, it should provide a comprehensive overview of their week, highlighting their accomplishments or setbacks. This profile should allow us to track their progress, or in some cases, any decline. Additionally, it should include vital information for the physiotherapist, such as whether the patient has experienced any medical incidents, hospital visits, urinary infections, falls, and other relevant details.*" (Physiotherapist#12, Web Platform)

Concerning the expansion of the exercise database, some physiotherapists recommended the inclusion of exercises that address the challenges often encountered by PwPD during ADLs (i.e., rolling over in bed, sit to stand transitions). In this line, one physiotherapist noted that more technical components should be added (i.e., rhythmical cueing, coordination elements) to ensure a more comprehensive exercise-based intervention. Conversely, another physiotherapist outlined the therapeutic value of enjoyable and relaxing activities, particularly for PwPD who encounter more difficulties with successful engagement in physical activity. Overall, physiotherapists found the exercises of the Web Platform suitable for a home-based setting. Nevertheless, ensuring patient safety was considered of utmost importance, requiring that all exercises adhere to safety standards. An example was provided regarding exercises in sitting positions, where the chair should be leaning against a wall since PwPD are more likely to push the chair backwards. The supervision of CGs was also deemed relevant for ensuring this aspect. In this vein, the perspectives of some physiotherapists follow:

"*Patients often encounter challenges in tasks such as rolling over in bed, covering and uncovering themselves with sheets, and transitioning from lying down to sitting or vice versa. These are common difficulties, and I believe addressing them could be valuable.*" (Physiotherapist#12, Web Platform)

"*I believe that some technical components are missing. Physiotherapists working with these patients often train for rapid movements, coordination, and multitasking skills.*" (Physiotherapist#13, Web Platform)

"*I was thinking about including a unique type of activity that might not fit the traditional exercise standards. This activity could be therapeutic by promoting physical activity in an enjoyable manner. The act of throwing a ball at a wall does not fit into categories like flexibility, strength, or balance; it likely falls within the neuro-motor domain. This domain, which is reported in the literature, involves the priming of cognitive skills. Throwing the same ball at a wall to the rhythm of music, creating a rhythmic experience, offers a different kind of therapeutic value.*" (Physiotherapist#9, Web Platform)

"*There is a crucial need to validate these exercises, ensuring maximum safety throughout the programs. For instance, when performing an exercise in a sitting position, the chair should ideally be placed against a wall. When seated, patients usually push through their upper limbs, extend their legs, and that increases the risk of the chair tipping backward.*" (Physiotherapist#1, Web Platform)

### Quantitative analysis findings

#### Assessment of the MoveOnParkinson conversational agent

The mean score for the 12 questions regarding the CA was 4.42/5.0 ± 0.79, ranging from 4.13 to 4.75. The mean score per participant was 4.44/5.0 ± 0.45, ranging from 3.58 to 5.00.

**Figure 2:**
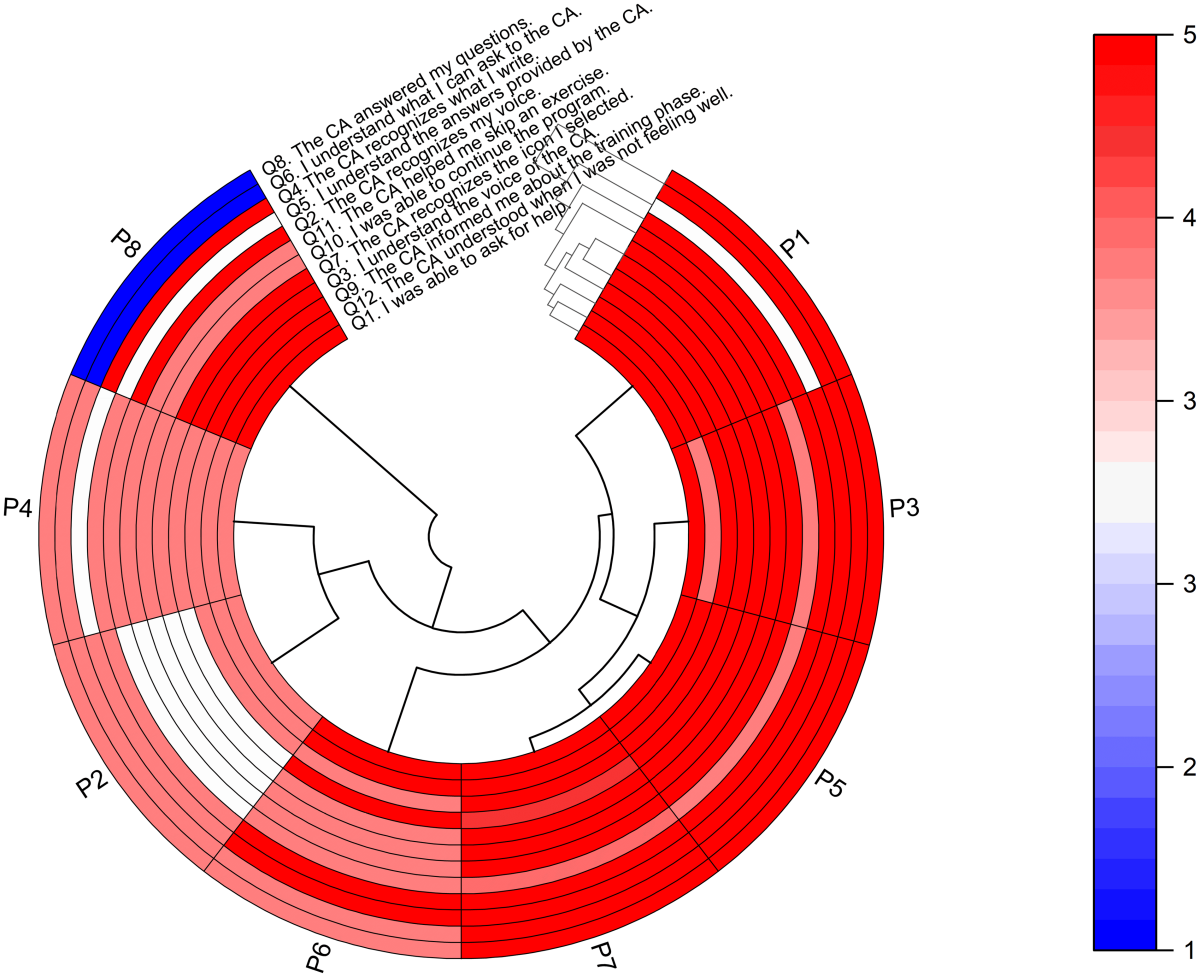
Heatmap with dendrogram showing hierarchical clustering of the eight participants (PwPD) into clusters, according to their responses on each of the 12 questions for assessing the CA. The red color indicates a higher score on a 1–5 Likert scale based on strength of agreement, and the blue color indicates a lower score. Q, Question; P, People with Parkinson’s Disease (PwPD).

In addition, a circular heatmap displaying individual responses of each participant for the CA assessment is presented (see Figure 2), with the *x*-axis representing the eight PwPD and the *y*-axis representing the 12 questions. The dendrogram offers insights into hierarchical clustering, revealing patterns of similarity in responses. Hence, the analysis suggests that four PwPD assigned higher average scores to the CA, followed by three participants who gave lower average scores. In line with these findings, the average score of each question suggests that PwPD were able to ask the CA for help, understanding its voice and the answers provided, while guiding the exercise program (i.e., indicating the training phase, exercise commands and prescription). Conversely, one participant rated the CA with the lowest score on questions 8 and 6, indicating dissatisfaction with the CA’s ability to address the specified questions and a lack of clarity regarding what could be asked of the CA within its specific domain of knowledge.

**Figure 3:**
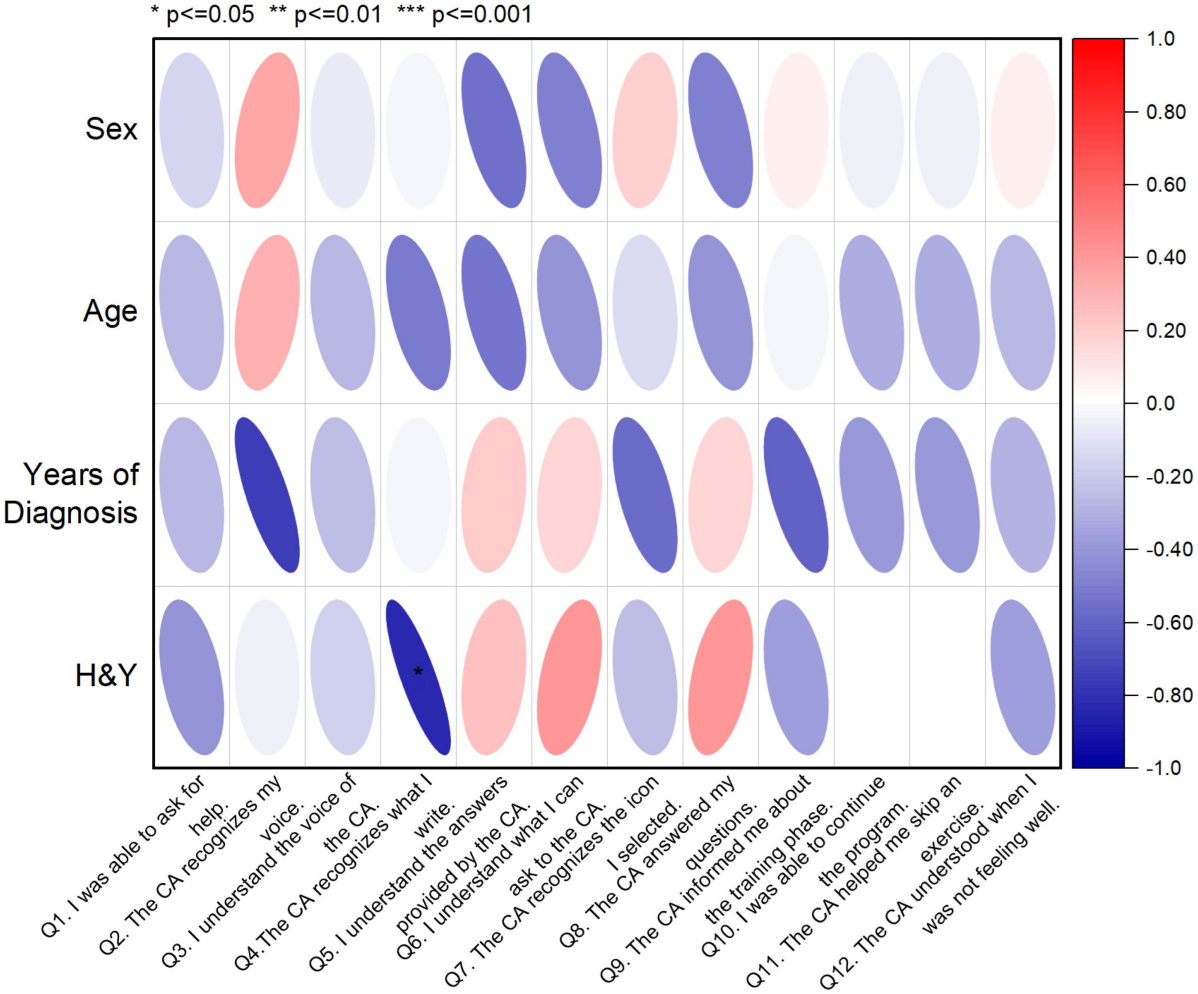
Correlation matrix displaying the relationships between SUS scores and socio-demographic data of PwPD sample (*n* = 8). The red color indicates a positive correlation, while the blue color indicates a negative correlation. Q, Question; H&Y, Hoehn and Yahr.

In addition, a correlation matrix was used to investigate the relationships between the SUS assessment of the CA and socio-demographic characteristics of PwPD (Figure 3) aiming to identify potential patterns and insights. The correlation coefficients were interpreted in terms of strength and direction. More specifically, from Figure 3, H&Y staging of PwPD shows a weak negative correlation with Q4, which indicates that higher PD severity has a negative impact on writing recognition from the *CA.* In all other combinations, no significant correlation was identified, showing lack of effect from the socio-demographics on the CA assessment variables.

#### Usability of the MoveOnParkinson web platform

Physiotherapists rated the MoveOnParkinson Web Platform, assigning it an average SUS score of 79.50 points with a standard deviation of ±12.40, falling within a range of 55–95. This mean score corresponds to an A-grade in usability, as it falls within the 85th to 89th percentile, which is indicative of a “Good” usability score. For the sample of 20 participants, a 95% confidence interval (CI) for the average SUS score was calculated using Student’s T distribution. The lower and upper bounds of the CI were found to be 73.70 and 85.30, respectively. This range corresponds to a B-grade (65th to 69th percentile) at the lower bound and an A+ grade (96th to 100th percentile) at the upper bound, as referenced in prior studies (61, 62).

**Figure 4:**
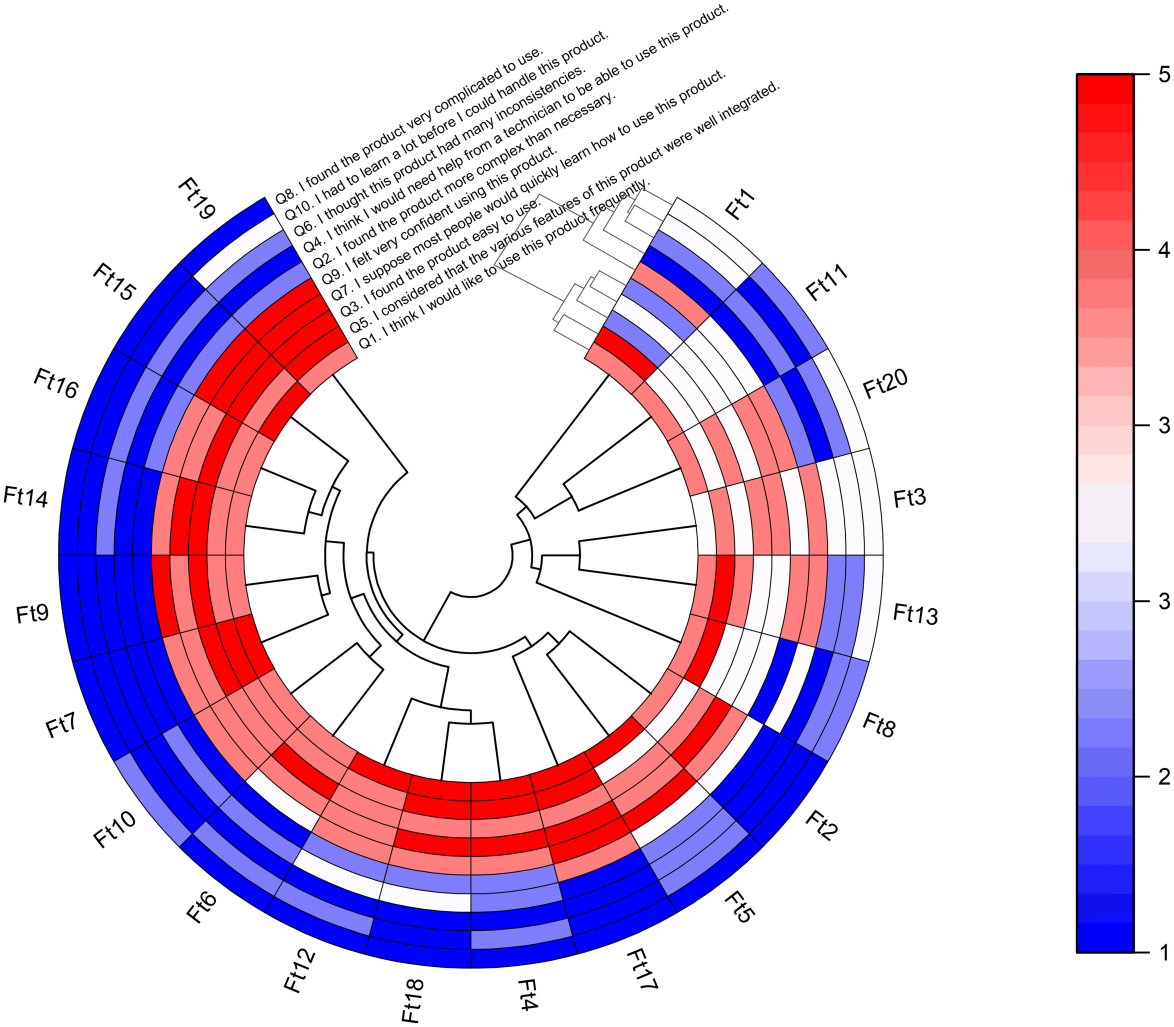
Heatmap with dendrogram showing hierarchical clustering of the 20 participants (physiotherapists) into clusters, according to their responses on each of the 10 SUS questions. For odd-numbered questions, the red color indicates a higher score on a 1–5 Likert scale based on strength of agreement, and the blue color indicating a lower score. The opposite scoring system applies for even-numbered questions. Q, Question; Ft, Physiotherapist.

The circular heatmap provides a visual representation of the individual SUS answers of each participant, where the *x*-axis represents the 20 physiotherapists, and the y-axis represents the 10 questions (see Figure 4). Additionally, the dendrogram offers insights into hierarchical clustering, revealing patterns of similarity in responses. Higher scores indicate better perceptions of usability on positive (odd-numbered) questions. However, Q3 and Q9 received lower scores from Physiotherapist# 1, which suggests that this participant did not find the MoveONParkinson Web Platform easy to use and did not feel very confident while using it. Conversely, lower scores indicate better perceptions of usability on negative (even-numbered) questions. Q2 received higher scores from Physiotherapists#1 and #13, indicating that they found the MoveONParkinson Web Platform more complex than necessary for its intended purpose. Q4 also received a higher score from Physiotherapist#1, which suggests that they believe that would need help from an expert to be able to use this interface. The analysis of Figure 4 also highlights the similarities on the answers of 14 physiotherapists, rating higher scores on the positive questions and lower scores on the negative questions, which translates into a higher SUS score. Despite giving lower ratings on the negative questions, Physiotherapists #8 and #11 shown a greater tendency to provide neutral scores, particularly on the positive questions.

**Figure 5:**
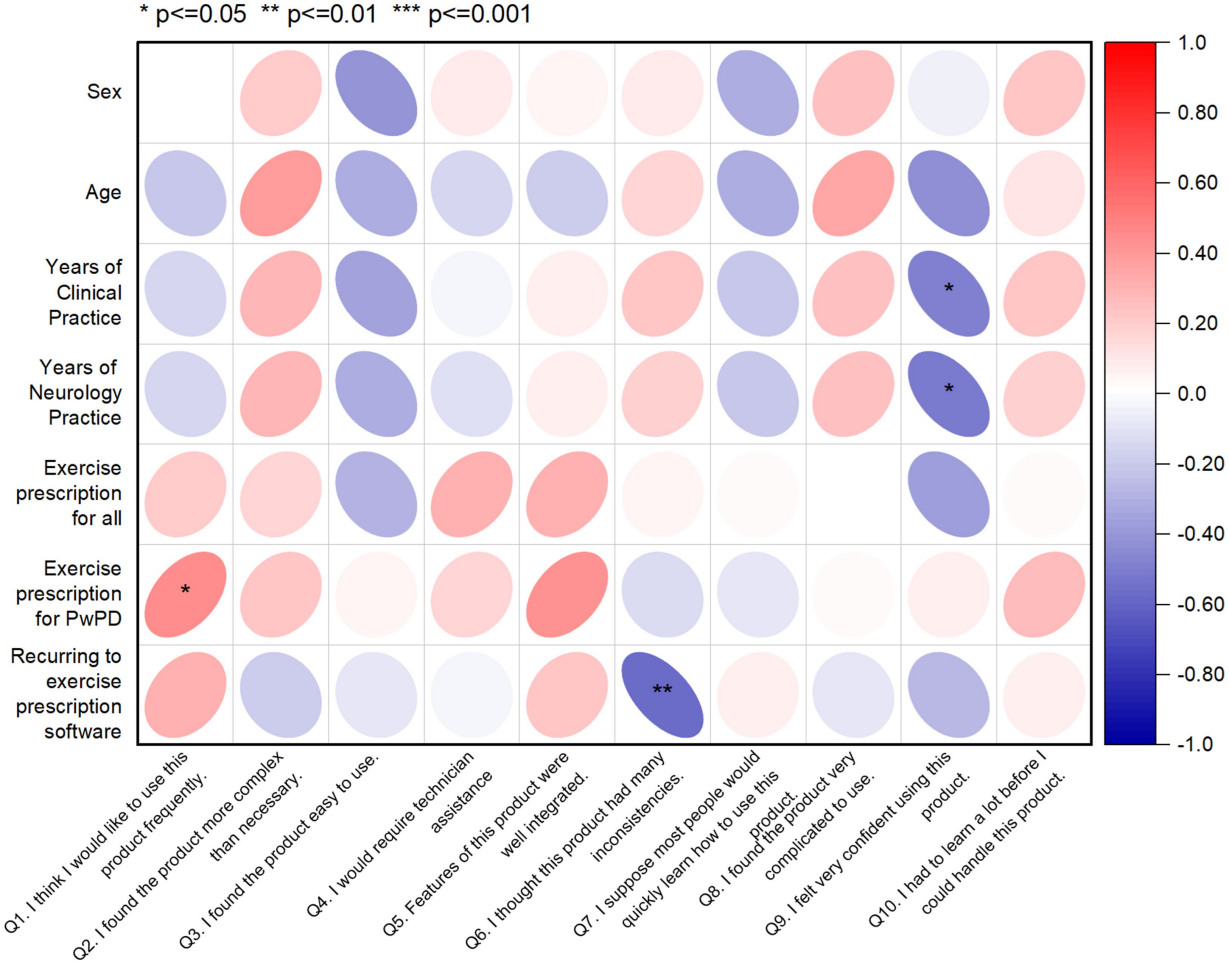
Correlation matrix displaying the relationships between SUS scores and socio-demographic data of the physiotherapist sample (*n* = 20). The red color indicates a positive correlation, while the blue color indicating a negative correlation. Q, Question.

In addition, a correlation matrix was used to investigate the relationships between SUS scores and socio-demographic characteristics of physiotherapists, aiming to identify potential patterns and insights (see Figure 5). The correlation coefficients were interpreted in terms of strength and direction. In this line, several noteworthy correlations emerged, shedding light on the influence of participant characteristics on their perceived usability of the MoveONParkinson Web Platform. A higher number of years of clinical practice, including in the field of neurology, shows a weak negative correlation with SUS question 9, indicating that more experienced physiotherapists felt less confident using the MoveONParkinson Web Platform. Moreover, a higher frequency of exercise prescription for PwPD shows a weak positive correlation with SUS question 1, which suggests that physiotherapists who prescribe exercise more often for PwPD would like to use this interface frequently. On the contrary, prior experience with exercise prescription software reveals a moderate negative correlation with SUS question 6. This suggests that physiotherapists who have used similar software in the past detected more inconsistencies within the MoveONParkinson Web Platform.

#### Integrating qualitative and quantitative findings

**Figure 6:**
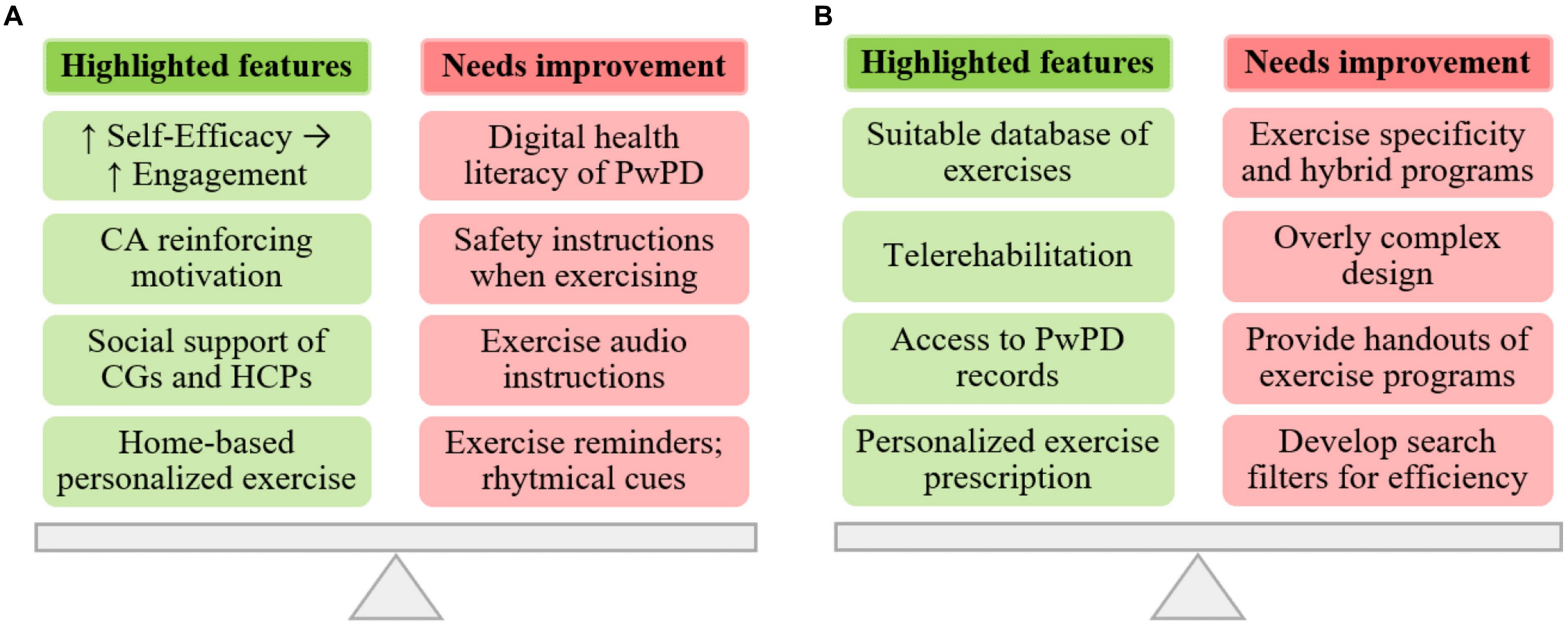
Integration of qualitative and quantitative findings regarding the MoveONParkinson interfaces: **(A)** Mobile App; and **(B)** Web Platform.

The integration of qualitative and quantitative findings was attained using a joint display, as shown in Figure 6. This display highlights the key features of the MoveONParkinson digital solution and presents recommendations for enhancing the corresponding interfaces. These recommendations are based on the insights gathered from both quantitative (i.e., SUS and CA assessment) and qualitative (i.e., semi-structured and thinking aloud interviews) data.

The perspectives of participants regarding the MoveONParkinson Mobile App emphasize this interface as an innovative solution for home-based personalized exercise. Moreover, reinforcing exercise self-efficacy through the features of the interface (i.e., motivational cueing provided by the CA) can positively influence exercise engagement, along with the social support provided by HCPs and CGs. The reported advantages of CGs involvement include the strengthening of the relationship between the dyad (i.e., CGs and PwPDs), health benefits for CGs deriving from increasing physical activity levels and facilitating access to the App. In this line, HCPs should ground their support in a collaborative approach that fosters reduced dependency on HCPs, encouraging active engagement of PwPD in their rehabilitation (i.e., goal setting). Nonetheless, some aspects were identified to further optimize this interface, namely the need to increase digital health literacy of PwPD (i.e., HCPs providing patient education on usage of the interface), reinforcing safety instructions of the exercises, enhance the quality of audio descriptions (i.e., decrease the pace of the narrator) on the exercise videos, providing rhythmical cues during some exercises, and adding reminders for performing the exercise programs.

In addition, physiotherapists outlined the innovative nature of the MoveONParkinson Web Platform, namely the availability of patients’ clinical and exercise related data, which enables physiotherapists to engage in personalized exercise prescription for PwPD. In this context, the exercise database, comprising over 200 exercises targeting strength, balance, and aerobic training, with options for progressions and regressions in difficulty, was found suitable and practical for facilitating exercise prescription for this specific population. HCPs also reported the relevance of this interface in the context of telerehabilitation, exemplifying the stages of transition from in-person care to a hybrid approach (i.e., complementing autonomous sessions with periodical in-person follow up). Conversely, recommendations for improving this interface include the need to add more specific exercises for PD (i.e., targeting difficulties in ADL’s), providing handouts of exercise programs as a complement to the MoveONParkinson App, enhancing the design of the interface and developing search filters for exercises and programs as a means of increasing navigation efficiency.

## Discussion

The MoveONParkinson digital solution is presented here as an innovative motivational approach for personalized exercise for PwPD. This study aimed to gather feedback and assess the acceptability of the MoveONParkinson Mobile App and the CA, and to gather feedback and assess the acceptability and usability of the Web Platform, using a mixed methods approach.

Through a qualitative approach, in-depth inductive thematic analysis of the interviews revealed three key themes, namely: Self-management, User Engagement, and Recommendations.

Theme 1 (Self-management) highlights the role of the MoveONParkinson digital solution on patient self-management. Physiotherapists outlined the need to engage in a collaborative approach to rehabilitation that supports patient empowerment. According to Collado-Mateo et al. (67), patient empowerment is achieved when these are able to deliberate and understand the most suitable course of action regarding their rehabilitation, after receiving adequate information and education. This can subsequently reduce dependence on HCPs and increase patient autonomy, which is one of the basic psychological needs to improve intrinsic motivation. Self-management interventions are educational interventions targeting cognitive processes and intrinsic motivation, designed to help people with chronic conditions to deal with its impact in everyday life. These interventions often include techniques of self-monitoring, problem solving, action planning, and goal setting (68). In fact, the latter has been identified as a key factor for increased adherence to the target behavior. This is aligned with current evidence suggesting that goals are more likely to be achieved when they are negotiated and agreed by patients and HCPs (67). Thereby, when adopting a self-management approach, HCPs recognize, foster and enhance this patient-held expertise, effectively empowering patients to be in control of their own lives (69).

Moreover, the Web Platform was considered a suitable resource to bridge the gap between in-person care and telerehabilitation, which aligns with its increasing demand since the beginning of the COVID-19 pandemic. In line with this, a systematic review conducted by Flynn et al. (70) found that home-based prescribed exercise improves balance-related activities and gait speed in PwPD, and these gains are similar to the ones obtained with equivalent center-based exercise. A study undertaken by Torriani-Pasin et al. (71) aiming to evaluate an asynchronous telemonitoring exercise program suggest that the program was safe, it had moderate adherence, and those exercising in the presence of their CG had higher attendance rate. These results not only make ground for using telerehabilitation as a resource to deliver safe and effective exercise programs, but also highlight the role of social support on engagement.

Theme 2 (User Engagement) emerging from thematic analysis comprises determinants (i.e., barriers and facilitators) of user engagement with the Mobile and Web interfaces. According to the perspectives of participants, facilitators for regular engagement with the Mobile App include concise exercise instructions, prior familiarity with the exercises, and being able to exercise at their own convenience with minimal equipment. These findings are consistent with the current evidence, which outlines the sense of accountability deriving from remote monitoring as facilitator for engagement (6, 72). In addition, the convenience of home-based interventions saves time and travel costs, which can be particularly advantageous in fostering autonomy and empowerment of PwPD without regular access to health services (72, 73).

Social engagement and social support, provided by CGs, family, peers, and HCPs, are perceived as important factors for reinforcing motivation and encouraging behavior change (6, 67). In this line, CGs involvement was considered beneficial for strengthening the relationship between the dyad. The current database of exercises includes several options for exercising in pairs, targeting balance, strength, and aerobic capacity. These exercises aim not only to increase patient motivation, but also to increase physical activity and exercise levels of CGs. Previous research suggests that CGs perceive their own involvement in rehabilitation approaches as natural (72). Nonetheless, providing informal care for PwPD can become a highly demanding endeavor. CG burden reflects the extent to which CGs perceive the adverse effects of providing care on several dimensions (i.e., emotional, social, physical, financial). Higher rates of CGs burden not only negatively affect their QoL but also have an adverse impact on the QoL of PwPD they care for, which leads to the worsening of health outcomes (74, 75). Therefore, the engagement of CGs must be thoughtfully considered, and they should receive consistent support, including education provided by HCPs.

Personal beliefs of low self-efficacy and low outcome expectations are additional barriers for sustained exercise engagement by PwPD (5, 6). Higher self-efficacy is a predictor of rehabilitation adherence, improves patient outcomes and reduces the financial burden of rehabilitation. Nonetheless, evidence suggests that self-efficacy can be positively influenced by HCPs (76). Identified strategies to improve self-efficacy should focus on improving performance accomplishment (i.e., mastery of experiences), promoting vicarious learning (i.e., learning from others), addressing emotional states (i.e., reducing negative emotional arousal) and providing useful verbal persuasion (34). The incorporation of these strategies and SCT constructs into the MoveONParkinson Mobile App could strengthen exercise self-efficacy, leading to behavior change toward exercise engagement.

As an interface aimed for exercise prescription, physiotherapists acknowledged the Web Platform as a useful resource to support PD management. The development of this interface was grounded by previous studies (24, 26, 27, 77–79) on the development of exercise-based digital interventions. In this line, several features were incorporated, namely personalized exercise prescription, collaborative goal setting, regular performance feedback and positive reinforcement. Nonetheless, physiotherapists’ engagement with the MoveONParkinson Web Platform can be hindered by the initial time investment to familiarize themselves with the exercise database and the features of the interface. Despite physiotherapists’ skills and knowledge, barriers such as lack of time limit their ability to provide tailored exercise programs according to patients’ expectations, motivations, and goals (75). This aligns with the reported need for an optimal design targeting greater efficiency. These barriers must be addressed since the MoveONParkinson digital solution will not be viable without the involvement of physiotherapists. In addition, current policies and practices of rehabilitation settings were considered an obstacle for broader implementation of MoveONParkinson. Thus, stakeholders’ engagement is required since physiotherapists are remarkably well positioned to meet the needs of people with chronic diseases regarding health behaviors and self-management capabilities (36).

Theme 3 (Recommendations), derived from thematic analysis is related with the recommendations for improvement the MoveONParkinson interfaces. In regard to the Mobile App, the main recommendations comprise providing complementary images of the exercises and clearer audio on video demonstrations. These perspectives align with reported obstacles for regular use of mHealth apps (i.e., unclear information) (67, 80). In addition, the incorporation of relatable metrics (i.e., daily steps) within the App as goals to achieve was also suggested. Being the decline in motivation a central barrier for exercise engagement, this strategy could be actionable in terms of increasing exercise and physical activity levels. This recommendation also aligns with current evidence (12, 25) for developing mHealth apps to support behavior change on PwPD. Overall, several recommendations provided by usability studies on mHealth were discussed and implemented on the MoveONParkinson Mobile App. For instance, the evaluation of a mHealth app to support PD management attained a high usability score (mean SUS of 84.4) (19).

Concerning the Web Platform, physiotherapists suggested the possibility to create multimodal exercise programs. These perspectives are aligned with the literature, supporting the effectiveness of multimodal exercise programs on improved patient outcomes (45, 47–49). Clinical fluctuations (i.e., “off” periods), comorbidities and fatigue can place further challenges to exercise participation (72). In line with this, being able to access patients’ clinical and exercise records was deemed paramount to better tailor exercise programs in accordance with their clinical status. Several physiotherapists offered their insights on additional exercises that could be incorporated into the database to ensure a comprehensive approach to rehabilitation. Thereby, recommendations included the addition of rhythmic cueing for some exercises, since this training modality appears to be effective on improving of gait velocity, stride length, and motor symptoms (81). The development of exercises based on the challenges encountered during ADLs was also suggested, which is aligned with updated rehabilitation guidelines for PwPD (45, 47–49). Moreover, patient safety was underlined as a major concern for unsupervised exercise. The risk-to-benefit ratio must be carefully considered when prescribing home-based exercise, of higher intensity or with minimal supervision (29).

Regarding the quantitative findings, the assessment of the CA resulted in a mean score of 4.42 ± 0.79. Overall, PwPD were able to ask the CA for help, understanding its voice and the answers provided, while guiding the exercise program. Conversely, greater disease severity was found to have a negative impact on the writing recognition feature. The variability of the mean scores per participant suggest that these results may not only be affected by the features of the CA, but also by the variability among end-users, which is aligned with the literature on characteristics of health interventions (82). The first report of using a CA on PwPD suggested it could be used to measure voice and communication outcomes, gathering information about challenges encountered, and provide education and support to the user (83). Nonetheless, current literature on the design and use of CAs for rehabilitation of adults with brain-related neurological conditions is heterogeneous and represents early stages of development and testing (84). Since the MoveONParkinson Mobile App is currently in an earlier stage of development, its usability was not evaluated using the SUS. Nonetheless, the perspectives of participants provide positive remarks on subjective measures of usability (i.e., perceived ease of use and perceived usefulness), which are related to user satisfaction (85).

Finally, the usability of the MoveONParkinson Web Platform was evaluated, focusing on the interaction between the user and the required task within a controlled environment, aiming to identify limitations of the interface (82). The MoveONParkinson Web Platform was attributed a mean SUS score of 79.50 (±12.40) by physiotherapists. In addition, both the upper and lower bounds of the 95% CI (73.70–85.30) are above the median score of 68 and the acceptability threshold of 70 (62). These results are aligned with recent findings, reporting a mean SUS score of 76.64 ± 15.12 for all the collected digital health apps, and of 83.28 ± 12.39 for “physical activity” apps (63). Conversely, 25% of the individual SUS scores were below the median score, and more experienced physiotherapists felt less confident using the MoveONParkinson Web Platform. The variability between individual scores is corroborated by the literature since the SUS has significant sensitivity to independent variables (61), and usage behavior is affected by the characteristics of health interventions and personal traits of its users (82).

### Limitations

As limitation of the study, we acknowledge the use of a purposive sampling method for participant recruitment due to the possible influence on the generalizability of these results, since these participants may have had a greater predisposition toward mHealth solutions. Furthermore, limitations may emerge regarding sample size. Increasing the sample size could help diversity representation and decrease any bias from outliers, providing generalization power to the findings. This could have potentially been influenced not only by a brief recruitment period, but also due to the methods used for data collection. While data collection for physiotherapists were conducted online, PwPD required an in-person setting, where patients’ affluence was also lower during the post-pandemic context. The use of the MoveONParkinson app in a home setting was not considered in this study; however, all participants were able to use independently both interfaces. Apparently, more extended conclusions could be drawn from the use of the MoveONParkinson app in-the-wild settings, provided the variability across different home settings. We anticipate a follow up study to further proceed with the testing and evaluation of the proposed app in-the-wild. In addition, using self-reported instruments to assess acceptability and usability of the interfaces could have influenced the results due to a potential social desirability bias, inherent to self-reported instruments. These limitations can be addressed by implementing a more extensive and comprehensive recruitment process, allowing participation in various stages of development, in line with an iterative design approach. Moreover, future studies can enhance methodological robustness by integrating objective outcome measures, such as success rates in completing digital solution tasks and ensuring formal validation procedures for assessment tools.

### Implications and future work

The findings from this study suggest that both the MoveONParkinson Mobile and Web interfaces are well-accepted, with the latter showing notably good usability. These observations align with the principles of the TAM, which encompass key constructs influencing users’ intentions to utilize and adopt new technologies (49, 83). In this context, a recent study has presented evidence supporting the effectiveness of a modified TAM framework in capturing users’ viewpoints related to the PROTEIN app (45). This AI-powered smartphone application offers personalized recommendations aimed at encouraging behavior change, specifically toward healthier lifestyles by enhancing dietary and physical activity habits (86). Hence, the relevance of these findings is substantiated by the need of high user retention rates of the interfaces as a foundation for achieving behavior change. As further practical implications, these findings indicate the potential for successful implementation of tailored home-based exercise programs. The relevance of this approach on the development of self-management skills through patient empowerment and active engagement in rehabilitation was also outlined. Moreover, the perspectives of physiotherapists and the usability scores attributed to the Web Platform showcase the role of efficient interface design, as proved by the specific tools and methods employed in our study. In this context, the keys to successful implementation and sustained user engagement lie in simplicity, clear instructions, and user-friendliness.

Both qualitative and quantitative results highlight the relevance of HCPs in guiding PwPD on effective usage of a mHealth intervention. In particular, physiotherapists are remarkably well placed for reinforcing behavior change by providing patient education (i.e., user interaction with mHealth technologies, informing about the benefits of regular exercise) (6, 67). A study undertaken by O’Brien et al. (75), aiming to assess the perceptions of physiotherapists on physical activity counseling and exercise prescription outlined their interest in being provided educational training. According to Speelman et al. (78), this training must emphasize the implementation of behavioral strategies and information to help PwPD overcome their barriers for exercise engagement. In line with this, Nimwegen et al. (87) recommend physiotherapists’ education on behavioral theories for successful implementation of behavior change interventions. Moreover, physiotherapists must formulate specific examples of exercise goals and being more attentive to patients with co-morbidities, cognitive dysfunction, and a lack of motivation. Hence, developing training programs for HCPs can be a valuable measure to provide them with the resources needed to better engage in patient education. Future work can be grounded by the theoretical frameworks underpinning this study (i.e., IDEAS, SCT). Thereby, optimization of MoveONParkinson would comprise the integration of end-users’ feedback gathered in this study, aiming to better suit their needs and preferences. This iterative refinement process will be further underpinned by a recent European research and innovation project “AI-PROGNOSIS: AI-based Parkinson’s disease risk assessment and prognosis,” 2023–2027.^1^ This project aims to advance PD diagnosis and care through novel predictive models combined with digital biomarkers from everyday devices, such as smartphones. To achieve this, AI-PROGNOSIS intends to identify user needs and effectively engage with key stakeholders to steer research questions and co-create the AI-PROGNOSIS toolkit for PD. Taking into account this exemplary model, aspects in need of improvement regarding the adoption and interaction with digital technologies would be identified. Hence, these results could guide decision making when optimizing MoveONParkinson to further meet the needs of end-users, aiming to increase engagement and to reinforce its elements of behavior change.

Once attaining intervention refinement, a small-scale evaluation would be undertaken, aiming to evaluate a signal of efficacy of MoveONParkinson on behavior change. These results would make ground for evaluating its effectiveness on behavior change and influence on QoL and PD symptoms. In this line, future research should include CGs in their sample profile. Involving CGs acknowledges their perspectives and unique insights on PD management that may not be captured from the sole perspective of PwPD. In fact, current evidence suggests a correlation between CGs involvement in research and improved patient outcomes (72, 74). Hence, by incorporating co-creation approaches and gaining a comprehensive understanding of the needs and experiences of CGs, we can effectively guide intervention development and develop strategies, such as educational content through the digital interface, aimed at alleviating CG burden. Finally, a more diverse sample of PwPD could be included in future studies, including variations in symptom severity and demographics, as this approach could yield valuable insights into customizing exercise programs to meet the diverse needs and contexts of different patient subgroups.

In conclusion, interpretation of these findings should consider its specific context and sample profile, which must be considered prior to generalizing the results of this study to broader settings. This underlines the need for further research and comparative studies to determine the broader applicability and external validity of these results.

## Data Availability

The raw data supporting the conclusions of this article will be made available by the authors, without undue reservation.

## Footnotes

^1^ https://www.ai-prognosis.eu/
