# Supplementary material for: MoveONParkinson: developing a personalized motivational solution for Parkinson’s disease management
Source: Front Public Health. 2024 Aug 19;12:1420171. doi: 10.3389/fpubh.2024.1420171 (PMC11366595; doi:10.3389/fpubh.2024.1420171)
Supplement: Supplementary file 1 [file Data_Sheet_1.DOCX]

| Patient Interview Guide Script | |
| --- | --- |
| 1 | What was your first impression of the app? |
| 2 | How would you describe your experience while using the app? |
| 2a | Did you find the app easy to use? Was it easy to follow instructions? |
| 2b | Did you find the information provided understandable? |
| 2c | What would be the positive and negative aspects of using the app? |
| 2d | Were there any screens with too much information? |
| 2e | Did you feel that the app is suitable for you? Why? |
| 3 | Do you have any recommendations to improve the app? |
| 4 | Would you consider using this app in the future? Why? |
| 5 | Is there anything else you would like to add or rephrase about the topics that we have covered? |

| Physiotherapist Interview Guide Script | |
| --- | --- |
| 1 | Do you believe these exercises are practical and suitable for a home-based setting, without physiotherapist’s supervision? |
| 2 | What is your opinion regarding the diversity of the exercises? |
| 3 | Do you believe these exercises and programs can contribute to patient's autonomy while exercising? |
| 4 | What is your opinion on the potential for patient engagement with these exercises? |
| 5 | Do you believe these exercises are suitable and specific for PwPD? |
| 6 | Which positive and negative aspects did you find while using the Web Platform? |
| 7 | Do you have any recommendations for improving the Web Platform? |

## 
