# Supplementary material for: MoveONParkinson: developing a personalized motivational solution for Parkinson’s disease management
Source: Front Public Health. 2024 Aug 19;12:1420171. doi: 10.3389/fpubh.2024.1420171 (PMC11366595; doi:10.3389/fpubh.2024.1420171)
Supplement: Supplementary file 2 [file Data_Sheet_2.DOCX]

| Online Questionnaire for the Conversational Agent (CA) assessment | |
| --- | --- |
| 1 | I was able to ask the CA for assistance. |
| 2 | The CA recognized my voice. |
| 3 | I understood the voice of the CA. |
| 4 | The CA recognized what I wrote. |
| 5 | I understood the answers provided by the CA. |
| 6 | I understood what I could ask to the CA. |
| 7 | The CA recognized the icon I selected. |
| 8 | The CA answered my questions. |
| 9 | The CA informed me about the training phase. |
| 10 | I was able to continue the program after asking for assistance. |
| 11 | The CA helped me skip an exercise. |
| 12 | The CA understood when I reported not feeling well. |

## 
